# Colder Ambient Temperatures Influence Acute Onset Canine Intervertebral Disc Extrusion

**DOI:** 10.3389/fvets.2020.00175

**Published:** 2020-04-07

**Authors:** Marc A. Barandun, Stella Bult, Stanislas Demierre, Beatriz Vidondo, Franck Forterre

**Affiliations:** ^1^Division of Small Animal Surgery, Department of Clinical Veterinary Medicine, Vetsuisse Faculty, University of Bern, Bern, Switzerland; ^2^Tierarztpraxis Dalbe, Basel, Switzerland; ^3^Neurovet, Ecublens, Switzerland; ^4^Department of Clinical Research and Veterinary Public Health, Vetsuisse Faculty, Veterinary Public Health Institute, University of Bern, Bern, Switzerland

**Keywords:** canine, intervertebral disc, weather conditions, temperature, environmental

## Abstract

Canine intervertebral disc disease is one of the most common neurologic conditions in veterinary medicine but the influence of environmental factors thereon has not been fully investigated. Subjectively, there has been the impression of increased admissions of acute spinal cord injuries due to intervertebral disc extrusion during periods of colder temperatures. In the present retrospective study, the different weather conditions (temperature, precipitation, sunshine, humidity, and atmospheric pressure) during the acute onset of clinical signs and a lag period of 3 days prior to the occurrence of symptoms were analyzed. One-hundred-and-one client owned dogs from the meteorological region of the Lake Geneva were presented to two referral centers during the 6-year (2007–2012) study period. Chondrodystrophic dogs represented 65.3% of our population, with the French Bulldog (19.8%) and Dachshund (17.8%) being the most common breeds. Multivariable logistic regression analysis identified an increased occurrence of intervertebral disc disease during colder temperatures. Our results are congruent with those of human studies which have shown that lower ambient temperatures are associated with more pain and increased risk for muscle injuries. The interplay of endogenous (breed, anatomical characteristics) and exogenous (environmental) factors should be addressed in a larger cohort study.

## Introduction

Canine intervertebral disc disease (IVDD) is one of the most common neurological conditions and is one of the main focuses of neurosurgical research (1). The various causes of IVDD can be divided into endogenous and exogenous factors. Endogenous influences refer to genetic features of each animal, which then influence their anatomy and configuration. Exogenous aspects are environmental factors, which are very variable and difficult to analyze.

Multiple studies already identified anatomical features as endogenous risk factors. Macroscopic differences in the composition of the vertebral column, vertebral body, and intervertebral disc are important for the occurrence of canine IVDD as well as microscopic and histological differences in these structures (2–6). In addition to anatomical and breed related predispositions, controversy exists on the role of age, sex, body weight, body condition score, muscle mass, and activity in the development of IVDD (7–10). More recent studies identified certain body dimensions (Dachshund) (11), age within different breeds (small breed dogs) (12) and the coat color (Pekingese) as risk factors for the occurrence of neurologic symptoms (13).

Environmental and lifestyle factors have also been investigated previously [e.g., influence of diet (14) and activity (5)]. Further, a recent study in Dachshunds demonstrated that dogs with an increased level of exercise (>30 min per day) and dogs allowed to jump on and off furniture were less likely to develop IVDD (15).

The influence of weather conditions on various diseases (myocardial infarction, urolithiasis, platelet adhesiveness, rheumatism) has already been described extensively in human literature (16–21). In addition, there is a reported seasonal variation in the incidence of gastric dilatation volvulus in military working dogs (22), as well as an association between meteorological changes and episodes of colic in horses (23).

Anecdotally and in the discussion with various clinicians there has been the impression of increased cases of IVDD during periods of colder temperatures. Therefore, the purpose of this study was to investigate the association of weather conditions (temperature, precipitation, humidity, atmospheric pressure, and sunshine duration) with the occurrence of acute IVDD. Our hypothesis was that lower temperatures would be associated with more cases of acute intervertebral disc extrusions.

## Materials and Methods

### Medical Records Review

Medical records of consecutive dogs with IVDD presented to two referral centers between January 1st 2007 and December 31st 2012 were reviewed. Dogs were included in the study if they had a complete medical history with an acute and known onset of clinical signs prior to presentation, a magnetic resonance imaging (MRI) diagnosis consistent of intervertebral disc extrusion and the full work-up including physical and neurological exam was available as well as the client's postal code. The client's domicile had to be within the meteorological region of the Lake Geneva to be included. This zone was chosen for its well-defined area with a homogenous climate and a reasonable population density. Chronic cases (clinical signs for longer than 10 days) and cases with incomplete work-up were excluded from the study.

### Data Collection

Signalment including breed, age, sex, and body weight was obtained from the medical record of each dog. Severity of neurologic injury was graded according to the 5-point scale developed by Scott, whereby a score of 1 represented spinal hyperesthesia only and a score of 5 indicated para-/tetraplegia with absence of deep pain sensation (24). The anatomic localization along the spine according to cross-sectional imaging was noted as cervical (C1-C7), thoracic (T1-T10), thoracolumbar (T11-L2), and lumbar (L3-L7). Additionally, the day of onset of the first clinical sings as well as the day of admission to the hospital was recorded and the duration of signs was calculated for each dog. Dogs were included if the period from onset of clinical signs until presentation to one of the institutions was within 10 days.

The Swiss Meteorological Institution (Meteo Schweiz) provided the meteorological data for the defined meteorological area, Lake of Geneva, from the weather station in Nyon-Changins. Data obtained included daily average atmospheric pressure (hPa), daily average temperature (°C), daily total precipitation (mm), daily total sunshine (h), and daily average humidity (%) for each day during the 6 years of the study period.

### Statistical Analysis

Patient data were merged with the time series of meteorological data by the date of onset of symptoms (date of insult). A binary variable was created to indicate whether the day in the time series had had a case or not. Logistic regression models were then calculated with this binary variable as the outcome (case yes/no) and each of the weather variables as the explanatory variable. The year and the season were always included as additional explanatory variables. Season was defined as four northern meteorological seasons [spring: March 1 to May 30, summer: June 1 to August 31, autumn: September 1 to November 30, winter: December 1 to February 28 (February 29 in a leap year)]. In order to account for the fact that the weather influence might have been the weather in the previous days to the case, we created additional weather variables shifted 1, 2, or 3 days prior to the insult day. Additional logistic regression models were calculated using the binary case variable as the outcome and the lagged weather variables as the explanatory variables. Several combinations were tested and the simplest most significant model selected as the final model. All analyzes were performed in NCSS Statistical Software12, Version 12.0.10, East Kaysville, Utah, USA.

## Results

### Dogs

After the initial medical record review, 101 dogs were identified with an acute IVDD from January 1st 2007 through December 31st 2012 from the meteorological region of Lake Geneva. Forty-five (44.6%) dogs were presented to the Kleintierklinik of the University of Bern and 56 (55.4%) dogs to Neurovet in Ecublens. The study population consisted of equal numbers of males (52; 51.5%) and females (49; 48.5%), a median age of 6.2 years (range, 1.2–15.8 years) and body weight of 9.55 kg (range, 2.9–45 kg). Date of birth was not available for two dogs and body weight was absent for one dog. The sample included 20 (19.8%) French Bulldog; 18 (17.8%) Dachshund; 12 (11.9%), mixed breed; 7 (6.9%) Cocker Spaniel; 6 (5.9%) Coton de Tuléar; 4 (4.0%) Bichon frise; 3 (3.0%) each of Jack Russel terrier and Tibet-Spaniel; 2 (2.0%) each of Beagle, Border terrier, German Shepherd, Pyrenean Shepherd, and Yorkshire terrier; and one (1%) each of American Staffordshire terrier, Basset Hound, Bernese Mountain Dog, Border Collie, Chihuahua, Dalmatian, Golden Retriever, Brussels Griffon, Maltese, Pekingese, Pointer, Papillon, Poodle, Rottweiler Sealyham Terrier, Shih-Tzu, and Toy Poodle. According to Smolders et al. (25), 66 (65.3%) of dogs were classified as chondrodystrophic. Given that breeds French Bulldog and Dachshund constitute only ≤1% of the officially recorded dog population living in Switzerland (ANIS, Identitas AG, Bern, Switzerland), these breeds appear to be predisposed to this condition. A mild yearly increase in the number of cases presented was observed over the study period (Table 1).

**Table 1:** Monthly cases seen over the study period.

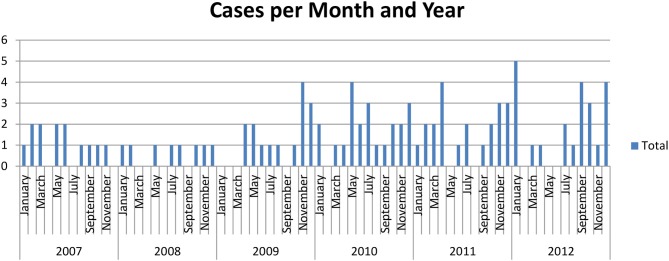

*Note that the there is a mild trend to increased case numbers per year over the 6 years (2007; 14 cases, 2008; 8 cases, 2009; 15 cases, 2010; 21 cases, 2011; 21 cases, 2012; 22 cases)*.

### Clinical and Diagnostic Findings

The anatomical localization of the lesion was lumbar in 43 (42.6%), thoracolumbar in 30 (30.0%), thoracic in 21 (20.8%) and cervical in 7 (6.9%) cases. At the time of admission, 40 (39.6%) dogs presented with ambulatory paresis (grade 2), 35 (34.7%) with a non-ambulatory paresis (grade 3), 17 (16.8%) with paralysis with presence of deep pain sensation (grade 4), 6 (5.9%) with only hyperesthesia without neurological deficits (grade 1), and 3 (3.0%) with paralysis with absent deep pain sensation (grade 5). The median time from the onset of clinical sings to presentation was 3 days (range, 0–10).

### Weather Conditions

Of all considered environmental variables, only temperature showed a mild but significant effect (Table 2). Days with a case of onset of clinical signs were less likely to occur with increasing temperatures (Odds Ratio ± 95%CI = 0.89 ± 0.80 – 0.90). This is congruent with our hypothesis of an increased occurrence of IVDD during colder days. Interestingly, this effect only appears when adding the lag variables, which, however, were individually not statistically significant. Other analyzed variables including atmospheric pressure, precipitation, sunshine and humidity or lagged variables did not demonstrate statistical significance. Models with chondrodystrophic dogs only did not converge, as there were not enough of these cases per month or season. In addition, days with cases were less likely in the year 2008 compared to 2012.

**Table 2:** Multivariable logistic regression model of the days with/without cases and the temperature at the day of clinical signs and temperature lagged 1, 2, or 3 days before onset of clinical symptoms, during each meteorological season compared to Winter over the 6 year study period.

**Independent variable**	**Odds ratio**	**Lower 95% CI**	**Upper 95% CI**	***P*-value**
Temperature	0.89	0.80	0.99	0.0347*
TempLag1	1.11	0.95	1.30	0.1760
TempLag2	0.94	0.80	1.10	0.4366
TempLag3	1.11	0.99	1.23	0.0729
Autumn	0.76	0.37	1.56	0.4537
Spring	0.65	0.31	1.35	0.2483
Summer	0.42	0.14	1.20	0.1042
2007	0.63	0.31	1.24	0.1813
2008	0.35	0.16	0.81	0.0137*
2009	0.67	0.34	1.32	0.2515
2010	0.98	0.53	1.82	0.9409
2011	0.94	0.51	1.75	0.8505

* *p < 0.05*.

## Discussion

Findings of the present study suggest that, besides the previously known/described effect of breed on IVDD (10), temperature might also have influenced the occurrence of IVDD in the Lake Geneva canine population from 2007 to 2012.

Chondrodystrophic dogs constituted just over 65% of our case population, which correlates well with what was previously reported in different studies (4, 26–28). The Dachshund, which was formerly found to be responsible for up to 79% of all IVDD cases (29), was the second most common breed in our study after the French Bulldog. The increased frequency of presentation of the French Bulldog for treatment of IVDD is in line with a general trend in Central Europe, corroborates well observations in other studies (30) and reflects the increasing popularity of the breed (31). The significant slight increase in cases in the years following 2008 is neither explained by a shift in breed representation nor by an increase in dog population. According to ANIS (Identitas AG, Bern, Switzerland) the number of dogs in Switzerland remained stable over the study period.

The median age of dogs at the time of presentation in our study was 6.2 years. This is consistent with numerous other publications, where disc extrusions commonly occurred between 3 and 7 years (32, 33). Moreover, 50-68.7% of discs have already undergone degenerative changes by the age of 6–7 years (4, 7, 15, 29, 34, 35).

Controversy exists in the literature regarding whether there is a sex predilection for IVDD (7, 8, 10, 15). In our study, both males and females were equally distributed (51.5% male, 48.5% female), which correlates with recent studies (15). Gonadectomy has been suggested to play a role in development of IVDD in Dachshunds especially when performed before 12 months of age (36), but was not assessed in our study. On the basis of the retrospective nature of the study, testing for dog characteristics is out of the scope of our study. Similarly, evaluating for different types of IVDD (e.g., Hansen type 1, acute non-compressive nucleus pulposus extrusion and hydrated nucleus pulposus extrusion) is not possible with our study design.

The most frequent localization of the IVDD was in the lumbar spine (42.6%) followed by the thoracolumbar (29.7%), thoracic spine (20.8%), and cervical (6.9%). In recent literature, thoracolumbar disc extrusion is reported to represent up to 80% of all cases (29, 37, 38). Nevertheless, this localization does not appear to be associated with patient outcome, apart from the cervical spine (38).

The assessment of different weather conditions on the occurrence of IVDD is a challenging task. The mild effect of temperature on the occurrence of IVDD observed in our study is similarly seen in some human studies pertaining to a variety of diseases, with lower ambient temperatures associated with more pain with disorders such as osteoarthritis, pelvic pain syndrome and musculoskeletal pain (39–41) and can also increase the risk for muscle injuries (42). However, in contrast to our findings, a comparable human study failed to show any correlation between the rate of hospital admission for back pain and different weather condition (43). Cold temperatures could affect the mobility of the musculoskeletal system in our patients as well, notably the spinal column. With a reduced mobility and less resistance to strain injuries, the biomechanical forces could eventually act differently or more traumatically on the spine, although this is purely speculative. Whether it is only the outside temperature which impacts the development of clinical signs, or rather the difference between outside and room temperature during colder seasons remains unknown. Direct sunlight could have a positive influence on dogs as it correlates with warmer outside temperatures. It leads to an increase in local skin temperature and muscle perfusion, which could increase the spinal mobility. Even though they appear to play a role in human diseases and back pain (39, 44, 45), none of the other weather variables showed any significant effect in our study. This could be due to small effect of those variables on the biomechanics of the different tissues as we only assessed the onset of IVDD signs.

The Lake Geneva region provided a large area with comparable meteorological situations in our study to collect our data from its dense surrounding settlements. At the same time, the lake by itself reduces rapid temperature changes and allows for less extreme weather conditions (46). Hence, the effect of different weather conditions on the occurrence of IVDD could have been reduced/underestimated.

We cannot exclude that some dogs included in the study may have experienced onset of clinical signs in another geographic and therefore meteorological area before being referred to one of our institutions. This information could have been missed at the time of anamnesis or not recorded in the medical record. Due to the geographical features of this area with national borders and more remote mountain regions adjacent to the lake, it seemed less likely to have a significant impact on the results of our study.

We only evaluated the weather conditions 3 days prior to and on the day of onset of clinical signs. As IVDD is considered a chronic disease with an acute onset of clinical signs, reflected in the inflammatory pattern in the extruded disc material (47), 3 days seemed like a reasonable criterion. Due to sample size limitations, our study did not take weather and environmental variations within a single month into account. For the future, a large cohort study including both cases and healthy subjects followed over a longer time period would be the next logical step to provide an estimate of incidence of the disease and to jointly analyse both the effect of environmental factors and dog characteristics such as breed, exercise, and age. In addition, further studies should consider a wider geographic area, as our study might be only representative for the Lake Geneva area.

## Data Availability Statement

The datasets generated for this study are available on request to the corresponding author.

## Ethics Statement

Ethical review and approval was not required for the animal study because of the epidemiologic retrospective nature of the study. Written informed consent for participation was not obtained from the owners because of the epidemiologic retrospective nature of the study.

## Author Contributions

MB: study design, data collection, statistical analysis, and generation of manuscript. SB: study design and data collection. SD and FF: study design and correction of manuscript. BV: study design, statistical analysis, and correction of manuscript.

### Conflict of Interest

The authors declare that the research was conducted in the absence of any commercial or financial relationships that could be construed as a potential conflict of interest.

## References

[1] Braund KG Canine intervertebral disc disease. In: Bojrab MJ, editor. Pathophysiology of Small Animal Surgery. Philadelphia, PA: Lea & Febiger (1981). p. 739–46.

[2] King AS The anatomy of disc protrusion in the dog. Vet Rec. (1956) 68:939–51.

[3] King AS Smith RN A comparison of the anatomy of the intervertebral disc in dog and man: with reference to herniation of the nucleus pulposus. Br Vet J. (1955) 111:135–49. 10.1016/S0007-1935(17)47302-7

[4] Hansen HJ. A pathologic-anatomical study on disc degeneration in dog, with special reference to the so-called enchondrosis intervertebralis. Acta Orthop Scand Suppl. (1952) 11:1–17. 10.3109/ort.1952.23.suppl-11.01 14923291

[5] Hoerlein BF Intervertebral disc protrusions in the dog. I. Incidence and pathological lesions. Am J Vet Res. (1953) 14:260–9. 13050890

[6] Bray JP Burbidge HM. The canine intervertebral disk: part one: structure and function. J Am Anim Hosp Assoc. (1998) 34:55–63. 10.5326/15473317-34-1-55 9527431

[7] Priester WA Canine intervertebral disc disease - Occurrence by age, breed, and sex among 8,117 cases. Theriogenology. (1976) 6:293–303. 10.1016/0093-691X(76)90021-2

[8] Goggin JE Li AS Franti CE. Canine intervertebral disk disease: characterization by age, sex, breed, and anatomic site of involvement. Am J Vet Res. (1970) 31:1687–92. 5528338

[9] Hoerlein BF Comparative disk disease: man and dog. J Am Anim Hosp Assoc. (1979) 15:535–45.

[10] Verheijen J Bouw J. Canine intervertebral disc disease: a review of etiologic and predisposing factors. Vet Q. (1982) 4:125–34. 10.1080/01652176.1982.9693852 6755879

[11] Levine JM Levine GJ Kerwin SC Hettlich BF Fosgate GT. Association between various physical factors and acute thoracolumbar intervertebral disk extrusion or protrusion in Dachshunds. J Am Vet Med Assoc. (2006) 229:370–5. 10.2460/javma.229.3.370 16881827

[12] Hakozaki T Iwata M Kanno N Harada Y Yogo T Tagawa M. Cervical intervertebral disk herniation in chondrodystrophoid and nonchondrodystrophoid small-breed dogs: 187 cases (1993-2013). J Am Vet Med Assoc. (2015) 247:1408–11. 10.2460/javma.247.12.1408 26642135

[13] Chai O Harrosh T Bdolah-Avram T Mazaki-Tovi M Shamir MH. Characteristics of and risk factors for intervertebral disk extrusions in Pekingese. J Am Vet Med Assoc. (2018) 252:846–51. 10.2460/javma.252.7.846 29553897

[14] Nipko R Ashmead D. Canine disc disease: cause, prevention, and a new approach to treatment. Vet Med Small Anim Clin. (1977) 72:1337–42. 242882

[15] Packer RM Seath IJ O'Neill DG De Decker S Volk HA. DachsLife 2015: an investigation of lifestyle associations with the risk of intervertebral disc disease in Dachshunds. Canine Genet Epidemiol. (2016) 3:8. 10.1186/s40575-016-0039-8 27826450 PMC5097381

[16] Freeman JW McGlashan ND Loughhead MG. Temperature and the incidence of acute myocardial infarction in a temperate climate. Am Heart J. (1976) 92:405–7. 10.1016/S0002-8703(76)80124-X 949035

[17] Elliott JP Gordon JO Evans JW Platt L. A stone season. A 10-year retrospective study of 768 surgical stone cases with respect to seasonal variation. J Urol. (1975) 114:574–7. 10.1016/S0022-5347(17)67085-X 1235381

[18] Klein E Jacobi E Hagemann G Kuhnke W. Effect of weather on platelet adhesiveness. Dtsch Med Wochenschr. (1973) 98:1189–90. 4711892

[19] Fujita K. Effect of weather on the incidence of urinary stone colic. Jpn J Nephrol. (1987) 29:1123–7. 3694890

[20] Rose MB. Effects of weather on rheumatism. Physiotherapy. (1974) 60:306–9. 4549520

[21] Al-Dabbagh TQ Fahadi K. Seasonal variations in the incidence of ureteric colic. Br J Urol. (1977) 49:269–75. 10.1111/j.1464-410X.1977.tb04135.x 912251

[22] Herbold JR Moore GE Gosch TL Bell BS. Relationship between incidence of gastric dilatation-volvulus and biometeorologic events in a population of military working dogs. Am J Vet Res. (2002) 63:47–52. 10.2460/AJVR.2002.63.47 16206779

[23] Barth R. Effect of weather on susceptibility of horses to colic. Tierarztl Prax. (1982) 10:203–8. 7179254

[24] Scott HW. Hemilaminectomy for the treatment of thoracolumbar disc disease in the dog: a follow-up study of 40 cases. J Small Anim Pract. (1997) 38:488–94. 10.1111/j.1748-5827.1997.tb03303.x 9403807

[25] Smolders LA Bergknut N Grinwis GCM Hagman R Lagerstedt A-S Hazewinkel HAW. Intervertebral disc degeneration in the dog. Part 2: Chondrodystrophic and non-chondrodystrophic breeds. Vet J. (2013) 195:292–9. 10.1016/j.tvjl.2012.10.011 23154070

[26] Ferreira AJA Correia JHD Jaggy A. Thoracolumbar disc disease in 71 paraplegic dogs: influence of rate of onset and duration of clinical signs on treatment results. J Small Anim Pract. (2002) 43:158–63. 10.1111/j.1748-5827.2002.tb00049.x 11996392

[27] Laitinen OM. Surgical decompression in dogs with thoracolumbar intervertebral disc disease and loss of deep pain perception: a retrospective study of 46 cases. Acta Vet Scand. (2005) 46:79–85. 10.1186/1751-0147-46-79 16108215 PMC2202789

[28] Lappalainen AK Mäki K Laitinen-Vapaavuori O. Estimate of heritability and genetic trend of intervertebral disc calcification in Dachshunds in Finland. Acta Vet Scand. (2015) 57:78. 10.1186/s13028-015-0170-7 26597811 PMC4656185

[29] Ito D Matsunaga S Jeffery ND Sasaki N Nishimura R Mochizuki M. Prognostic value of magnetic resonance imaging in dogs with paraplegia caused by thoracolumbar intervertebral disk extrusion: 77 cases (2000-2003). J Am Vet Med Assoc. (2005) 227:1454–60. 10.2460/javma.2005.227.1454 16279391

[30] Klesty A Forterre F Bolln G Postoperatives Ergebnis bei Diskopathien des Hundes in Abhängigkeit von Rasse, Lokalisation und Erfahrung des Chirurgen: (1113). Fälle Tierärztl Prax Ausg K Kleintiere Heimtiere. (2019) 47:233–41. 10.1055/a-0948-9187 31434123

[31] Jones BA Stanley BJ Nelson NC. The impact of tongue dimension on air volume in brachycephalic dogs. Vet Surg. (2019) 49:512–20. 10.1111/vsu.13302 31361346

[32] Aikawa T Fujita H Kanazono S Shibata M Yoshigae Y. Long-term neurologic outcome of hemilaminectomy and disk fenestration for treatment of dogs with thoracolumbar intervertebral disk herniation: 831 cases (2000-2007). J Am Vet Med Assoc. (2012) 241:1617–26. 10.2460/javma.241.12.1617 23216037

[33] Bergknut N Egenvall A Hagman R Gustås P Hazewinkel HAW Meij BP. Incidence of intervertebral disk degeneration-related diseases and associated mortality rates in dogs. J Am Vet Med Assoc. (2012) 240:1300–9. 10.2460/javma.240.11.1300 22607596

[34] Brown NO Helphrey ML Prata RG Thoracolumbar disk disease in the dog: a retrospective analysis of 187 cases. J Am Anim Hosp Assoc. (1977) 13:665–72. 10.1111/j.1752-1688.1977.tb02007.x

[35] Rohdin C Jeserevic J Viitmaa R Cizinauskas S. Prevalence of radiographic detectable intervertebral disc calcifications in Dachshunds surgically treated for disc extrusion. Acta Vet Scand. (2010) 52:24. 10.1186/1751-0147-52-24 20398282 PMC2873269

[36] Dorn M Seath IJ. Neuter status as a risk factor for canine intervertebral disc herniation (IVDH) in dachshunds: a retrospective cohort study. Canine Genet Epidemiol. (2018) 5:11. 10.1186/s40575-018-0067-7 30459956 PMC6236875

[37] Olby N Levine J Harris T Muñana K Skeen T Sharp N. Long-term functional outcome of dogs with severe injuries of the thoracolumbar spinal cord: 87 cases (1996-2001). J Am Vet Med Assoc. (2003) 222:762–9. 10.2460/javma.2003.222.762 12675299

[38] Ruddle TL Allen DA Schertel ER Barnhart MD Wilson ER Lineberger JA. Outcome and prognostic factors in non-ambulatory Hansen Type I intervertebral disc extrusions: 308 cases. Vet Comp Orthop Traumatol. (2006) 19:29–34. 10.1055/s-0038-1632970 16594541

[39] Timmermans EJ Schaap LA Herbolsheimer F Dennison EM Maggi S Pedersen NL. The influence of weather conditions on joint pain in older people with osteoarthritis: results from the European project on osteoarthritis. J Rheumatol. (2015) 42:1885–92. 10.3899/jrheum.141594 26329341

[40] Hedelin H Jonsson K Lundh D. Pain associated with the chronic pelvic pain syndrome is strongly related to the ambient temperature. Scand J Urol Nephrol. (2012) 46:279–83. 10.3109/00365599.2012.669404 22452545

[41] Pienimäki T Karppinen J Rintamäki H Borodulin K Laatikainen T Jousilahti P. Prevalence of cold-related musculoskeletal pain according to self-reported threshold temperature among the Finnish adult population. Eur J Pain. (2014) 18:288–98. 10.1002/j.1532-2149.2013.00368.x 23881586

[42] Scott EEF Hamilton DF Wallace RJ Muir AY Simpson AHRW. Increased risk of muscle tears below physiological temperature ranges. Bone Jt Res. (2016) 5:61–5. 10.1302/2046-3758.52.2000484 26883967 PMC4852792

[43] Okwerekwu G Brooks F Spolton-Dean C Khurana A Manoj-Thomas A Cordell-Smith J Is there a seasonal variation of acute admissions for back pain. Spine J. (2015) 15:S76 10.1016/j.spinee.2014.12.111

[44] Fagerlund AJ Iversen M Ekeland A Moen CM Aslaksen PM. Blame it on the weather? The association between pain in fibromyalgia, relative humidity, temperature and barometric pressure. PLoS ONE. (2019) 14:e0216902. 10.1371/journal.pone.0216902 31075151 PMC6510434

[45] Kasai Y Takegami K Uchida A. Change of barometric pressure influences low back pain in patients with vacuum phenomenon within lumbar intervertebral disc. J Spinal Disord Tech. (2002) 15:290–3. 10.1097/00024720-200208000-00005 12177544

[46] Eichenlaub VL Lakes, effects on climate. In: Fairbridge RW, Oliver JE, editors. Climatology. Boston, MA: Springer (1987). p. 534–9. 10.1007/0-387-30749-4_103

[47] Monchaux M Forterre S Spreng D Karol A Forterre F Wuertz-Kozak K. Inflammatory processes associated with canine intervertebral disc herniation. Front Immunol. (2017) 8:1681. 10.3389/fimmu.2017.01681 29255462 PMC5723024

